# Is social exclusion pushing the Pakistani Hijras (Transgenders) towards commercial sex work? a qualitative study

**DOI:** 10.1186/1472-698X-12-32

**Published:** 2012-11-19

**Authors:** Muhammad Ahmed Abdullah, Zeeshan Basharat, Bilal Kamal, Nargis Yousaf Sattar, Zahra Fatima Hassan, Asghar Dil Jan, Anum Shafqat

**Affiliations:** 1 Shifa College of Medicine, Pitrus Bukhari Road, Sector H-8/4, Islamabad, Pakistan

**Keywords:** Hijras, Commercial sex work, Social exclusion, HIV and Pakistan

## Abstract

**Background:**

The *Hijra* is a distinct type of gender role in South Asia where men act like women. This group of people is socially excluded by the general community, in terms of attainment of an opportunity for a socially productive life. Often this sort of deprivation forces these individuals towards professions like sex trade, in pursuit of sustenance, which as a consequence places them as a key block in the puzzle of an impending generalized HIV epidemic in Pakistan.

**Methods:**

This study is a qualitative study, which involved 8 in-depth interviews and four focus group discussions, conducted in Rawalpindi and Islamabad (Pakistan) from February to April 2012. The data was audio taped and transcribed. Key themes were identified and built upon. The respondents were contacted through a gate keeper Hijra who was a member of the hijra community. Multiple interview sessions were conducted with each respondent.

**Results:**

Two key categories of the Hijras were identified as Khusrapan and Zananapan, during the in-depth interview sessions. This initial information paved way for the four focus group discussions. The data was presented using key themes which were identified. The study participants explained their life histories to us which made it obvious that they had been socially excluded at many stages of their lives from performing normal social functions. This lack of occupational and educational opportunities pushed them towards entering the risky business of selling sex.

**Conclusion:**

The transgender community is socially excluded by the Pakistani society which is leading them to indulge in commercial sex and putting their lives at risk. Prudent measures are needed to form community based organizations managed and led by hijra community and addressing their social exclusion and risky behaviors.

## Background

Commercial sex work is often referred to as the oldest profession in the world [1], and is a prevalent practice in most countries. Pakistan has a long history of sex work [2] and multiple population groups that are involved in bartering sex for financial benefits; these groups include female, male and hijra sex workers [3]. The national surveillance data suggests that every year over 60 million sex acts are sold to 3 million clients, in Pakistan [4]. Sex work is rarely a profession opted by choice, it involves many risks, not to mention the stigma and discrimination attached to the profession [5]. In a qualitative study conducted on the Female sex workers of Malawi, when asked; why they continued to practice unsafe sex in the presence of a full throttle HIV epidemic, a subject responded by saying that *“each business has its own risks”*[6].

In many parts of the world transvestites are involved in various commercial and non-commercial sexual activities and have thus been identified as key players in the spread of HIV and other Sexually Transmitted Infections. In South Asia, particularly in Pakistan, India and Bangladesh, the term ‘Hijra’ is often used, which is a distinctive form of gender role manifestation, where men act like women. The term Hijra is an umbrella term which includes various forms of gender deviances. They include true hermaphrodites, transgender / cross dressers homosexuals and bisexuals. Hijras are mainly classified into Khusras; who are true hermaphrodites, Zananas; who are merely cross dressers and Narbans; who are castrated men [3]. Many Hijras sell sex to earn a living and hence they are at a great threat of contracting and spreading various sexually transmitted diseases. To describe these individuals the term Hijra Sex Workers is in use; which describes them as those who identify themselves as hijra and indulge in sexual activity with another man for money or financial benefits [7]. According to the National AIDS Control Program (NACP) an estimated 23,317 HSW’s are selling sex in 14 major cities of Pakistan, out of which around 490 (2.1%) reside in Rawalpindi [8].

Selling sex has long been associated with stigma and discrimination [5], and when supplemented by homophobia the unacceptability increases even further [9]. The concept of ‘Social Exclusion’ is a pertinent issue with reference to the treatment of the Hijras in the Pakistani society [10]. Socially-excluded people or groups of people are not able to participate in societal mainstream activities. Factors contributing to social exclusion include poverty, non-dominant social identities, e.g. race, ethnicity, religion, and gender; social locations (migrants, refugees); demographic features (occupation, educational level); and health conditions, e.g. disability, stigmatized diseases, such as HIV and AIDS [11]. Social, economic, cultural and political aspects of exclusion enforce deprivations of the basic amenities of life [12]. Social exclusion has been defined in a number of ways, regarding multiple aspects of human behaviors and situations; the following definition by Beall and Piron seemed to be the most appropriate with regards to our study question: *“a process or state that prevents individuals or groups from full participation in social, economic and political life and from asserting their rights. It derives from exclusionary relationships based on power”*[12]. In a recent study conducted in the Hijra population of Bangladesh, Khan et al. presented a rich description of social exclusion of the hijra population in Bangladesh, arguing that, before prevention of HIV-transmission interventions can be effective broadly in this population, the underlying factors that drive the hijra to high risk behaviors must be addressed [13].

The evolution of Pakistan’s sex industry has already started having repercussions on the generalized community, as recently the HIV epidemic in Pakistan has entered the concentrated phase from a low prevalence phase [8], and with the involvement of the sex workers in the epidemiological trap, the country seems to be following the’ Asian epidemic model’ [14]. Recent data suggests that Hijras are a major source of spreading various STIs. In terms of HIV, sex between men is noteworthy because it can involve anal sex, which when unprotected carries a very high risk. At least 5-10% of HIV infections worldwide are estimated to occur through sex between men. As men who have sex with men may also have sex with women, if infected they can transmit the virus to their female partners or wives [15]. Although sex between men is often associated with an isolated HIV epidemic, it should also be regarded as related to the epidemic in the general population [16]. In a recent study conducted in the HSWs of Lahore it was found out that 69.5% HSW’s had never used condom [10]. In 2008, HIV prevalence was 27.6% among hijra sex workers in Larkana [17]. During 1999; Baqi et al. found that 37% of Hijras in Karachi were positive for syphilis [18]. National Study of Reproductive Tract and Sexually Transmitted Infections of high risk populations during 2004 showed that 60.2% Hijras in Karachi and 35.9% in Lahore were suffering from Syphilis, whereas 0.5% Hijras in Lahore and 1.5% in Karachi were HIV positive [19].

The present study purports to look into the lives of the Hijra Community of Rawalpindi in order to identify the factors that they think are pushing them into the vicious cycle of commercial sex trade. The question that we would like to pose to the reader is how excluding certain groups of people at different levels effects the society as a whole. In order to achieve this purpose a qualitative study approach was brought into use, for gaining access to the appropriate information.

## Methods

The quantitative component of the study included a questionnaire based survey. This study was carried out in various localities of Rawalpindi and Islamabad. The more prominent areas which provided us with the study samples included, Dhok Chiraghdin, Pindora Chungi, Service Road, Commercial Market (Rawalpindi) I-8 Markaz, F-10 Markaz (Islamabad). The component regarding STI awareness has been published elsewhere [20].

Once we had gathered and analyzed the data from the quantitative component of this study, the next logical question that grabbed our attention was that; *“why does the hijra community depend on commercial sex work to generate an income?”* In order to get pertinent answers to this query we decided to head to the field once again, this time with a qualitative approach. This time our research team comprised of two medical doctors with a focus on public health, a clinical psychologist, five medical students and a Hijra sex worker as a gate keeper. The public health approach was strengthened by the enthusiasm of the medical students, and the psychologist gave us an insight into the way people think. The gate keeper proved to be an integral part of the research team through his deep insight into the issues of the community under research. The qualitative component of this study was carried out mostly in the study population of Hijras from Rawalpindi, from February to April, 2012.

Step one included unstructured qualitative interviews with 8 respondents. Through purposive sampling men who identified themselves as Hijras and who were willing to share their experiences were approached by our gate keeper. Multiple meetings with the participants were held so that the researcher developed rapport with the participants. The interviews were conducted in private, with no family or community member around. This helped in breaking the ice and gave the participants a chance to re-think past events and remember other associated experiences that could be shared at subsequent meetings. The interviews were tape recorded with the participants’ consent and transcribed within two days by the interviewers. The researcher went back with the written narration for verification followed by any further probing identified as needed by the research team.

These interviews gave us the baseline investigation for our journey into the lives of the Hijras. In these interviews we found out that most Hijras follow two schools of thoughts namely the *“Zananapan”* and *“Khusrapan”.* On the basis of this baseline information we decided to proceed towards step two of our study which employed four focus group discussion sessions. Two sessions were conducted with two groups of 7 and 6 Hijras belonging to Zananapan school of thought respectively. Two other FGD’s were also carried out with 8 and 7 respondents belonging to Khusrapan school of thought respectively. Discussion with each group was conducted separately to avoid conflicting opinions. The respondents were questioned till saturation was achieved in the responses. The conversations were tape recorded, with the consent of the participants and transcribed. The transcripts were read over several times to gain better understanding of the information provided by the respondents. The concept of Social Exclusion described by Popay et al., in the report titled, “Understanding and tackling social exclusion: final report to the WHO Commission on Social Determinants of Health”, was kept in mind during data analysis [21].

### Ethical considerations

Ethical clearance for the study was sought from the Institutional Review Board of Shifa College of Medicine (Islamabad). Verbal consent from all the participants was obtained prior to their participation in the study, after the permission from the decision maker in the house (Guru ;The leader of each group of Hijras) was granted. The study questions could have raised emotional concerns for the participants, and hence the psychologist remained at hand to steer the conversations in a compassionate manner.

## Results

In the natural course of scientific investigation the Naturalistic approach through inductive reasoning is essential to study issues while considering person to person variations, for identifying and implicating causes. The present study was initiated by conducting in-depth interviews. These interviews provided us with the information that probed the need for further qualitative investigation through focus group discussions. The main themes identified in the interviews are provided in the following text (Table 1).

**Table 1:** Profiles respondents of In-depth interviews

**Number**	**Age**	**Identity**	**Status in social hierarchy**	**Age of first contact with Hijras**	**Age of first sexual encounter**	**Level of education**	**Source of income**
1	45	Zanana	Guru	14	12	Primary	Chelas
2	27	Khusra	Chela	15	20	High School	Dance and Alms
3	33	Zanana	Independent	14	9	Uneducated	CSW,Tailor,Dance
4	16	Zanana	Chela	15	14	Below Primary	CSW
5	42	Narban	Guru	10	10	Uneducated	CSW,Dance, Beggary
6	52	Khusra	Guru	Does Not Remember ^1^	Virgin	Home educated ^2^	Alms and Charity
7	21	Zanana	Chela	15	15.5	Secondary	CSW
8	68	Zanana	Guru	Does not remember	Does not remember	uneducated	Alms and charity

### Childhood and early life: realization of a difference

Contrary to the popular myth that gurus demand custody of a child born with ambiguous genitalia, most of these children spend their early childhood with their families till they start demonstrating inappropriate behaviors based on gender identity. At this point in time they start experiencing repercussions from their family members in the form of physical and psychological reprimands. As narrated by one of our respondents: *“Since I was a child I had a fascination with dolls, makeup, jewelry and wearing my sister’s clothes. I used to help my mother with the daily chores and she loved me the most for my polite nature, but my father and brothers often beat me up and made fun of my ways”.*

Hijras as children are many a times socially excluded by their own family members, who consider them as a cause of disgrace and stigmatization for the whole family. Many Hijras claim that they have *“a woman’s soul trapped in a man’s body”,* and this characteristic was identified by most respondents at a very early age. Most of the Hijras spend a disturbing childhood where the battle for an identity is always haunting their conscious and subconscious practices. Most of these children get home schooled but those who are fortunate enough to undergo formal schooling, end up as drop outs because of social and sexual victimization. As recalled by one hijra: *“I joined school when I was 5 or 6 years old. I was sent to an only boys school. I used to stay alone as I had no interest in the harsh games that the other boys played. Often the school guard showed compassion towards my loneliness. I always called him ‘chacha (paternal uncle)’, and respected him a lot. But one day he tried to make me touch his private parts, I ran home and told my father about it; who beat me and prohibited me from going to school”.*

### A new beginning: the world of hijras

A predominant number of our respondents were introduced to the Hijras way of life early in their teenage years. These encounters took place mostly at wedding and childbirth ceremonies where Hijras had been invited to dance for the entertainment of the guests. This open display of feminism by these apparently masculine looking people attracted most of the respondents towards their way of living. They considered it as a life style where they could freely search for a true gender identity and *“Be who they really are”.* Many respondents claimed that their personal encounters with the hijra community initiated through brief social visits paid to the residents of the hijra households in their vicinity. The accepting and affectionate attitudes of the Hijras proved to be a major pull factor for these young susceptible minds. As a respondent narrated*; “I used to visit the baitakh (residence) of the hijra group that lived two streets away from my house. Every time I went there they served me sweet meats and soft drinks, they dressed me up and put make up on my face. This was the best time I had while I was growing up. This world of the Hijras was where I found inner peace”.*

### Life as a hijra: away from the mainstream

The hijra way of life is governed by strict laws of social hierarchy, where the Guru is the head of the household, employing the role of a spiritual leader and influencing the lives of his Chelas (subordinates), in every way. The hijra household usually comprises of the guru at the top and five to ten Chelas who work and live under one roof. The guru usually enjoys privacy in his own room and is referred to as “*Ammi Jee (Mother)”* in most circumstances. The guru is the main decision maker of the family unit, all the Chelas are expected to provide their earnings to the guru who utilizes these finances for running the daily expenses of the household, in addition to providing the Chelas with pocket money. The main heads of expenditure are spent on house rent, electricity and gas bills and for transportation and preparation of functions. Some of the money is also spent on the entertainment of guests and clients. The guru achieves this social status of respect and dignity by giving up his sexual practices and on the basis of age and seniority. As one guru said that; *“I used to sell sex when I was young and attractive, but as I grew old and ugly no one desired me. We had a few Hijras in our group who wanted to become my Chelas instead of our old guru’s, so we moved to this new location where I am the guru now. I stopped having any more lovers because how can I lead my Chelas if I do the same work as them. Besides being the guru feels nice I get to pass orders which all my Chelas follow”.*

### Khusrapan vs. Zananapan: two schools of thought

During our initial interviews we got acquainted with the two predominant fashions in which different groups of Hijras function. The Khusrapan (Hermaphrodites) way of thinking was found in people who had a guru who was a true hermaphrodite. This group traced its roots to the early history of the Indo-Pak subcontinent. According to them they originated from the Khwajahsaraas of the Mughal dynasty. This group considered commercial sex work and beggary to be evils haunting their community, and thought of these practices as a menace to the exalted status, they once enjoyed in the society. The Khusrapan followers thought that it was inappropriate for them to indulge in sexual practices and only depended on finances generated through alms given by people in return for their prayers and blessings. One respondent stated that *“A real Khusra would never spread his hands or legs in front of anyone, all of these sex workers and beggars are imposters, ruining our good name”.*

On the other hand the Hijras belonging to the Zananapan school of thought had totally different views. In the hijra vocabulary Zananas are those men who are born with male bodies but choose to live and look like women. Most of them sell sex and perform beggary on the streets. According to a follower of Khusrapan; *“These men try to hide their homosexuality under the cover of being Khusras”.* The Zananapan folk think otherwise and claim that they are women at heart and because of the fact that the society shuns them they have no other option than to sell sex and beg people for a living. As one of the Zananas said that*;” I am a woman at heart and every woman desires love from a man”.*

### Living environments and social contacts

The Khusrapan school of thought enjoys a respectable place in the society; the local populations refer to them as *‘Baba’ (the respected one).* It is a common belief that as they are *“Incomplete beings”;* neither whole men nor women, they hold a very respectable place in the court of God almighty, and that their prayers are well heard. Also there is an element of fear associated with this group. There is a popular myth that if one is cursed by a hijra, there is nothing that can undo it. Because of their inconspicuous behavior and the fact that they mostly remain indoors, the Khusra group of hijra’s does not enjoy the amount of attention that the more flamboyant ‘Zananas’ attract, during their displays of exaggerated feminism, in their pursuit of financial benefit. Khusras dwell in confined communes, living mostly on alms and charity; which the surrounding population donates. Every household, no matter how poor they are, considers sharing their meals with them to be a great privilege. Households of these Khusras were beautifully decorated. Some had pictures and paintings depicting their exalted status in the Mughal Empire. A majority of Khusras lived in a family house, which was transferred down generation after generation; a few had paying guests as well.

Zananas on the other hand, live in commercial areas, mostly residing in single room dwellings in dingy back alleys. No one ventures into those areas except for innocent children, who have no idea about what’s going on there, and their clients. These localities were full of homeless drug addicts who resided in open spaces and slept on the streets at night. Many of the Zananas accepted the fact that they used recreational drugs once in a while, although only a small number claimed of trying injectable drugs. People in the locality seldom visited them, some of them even forbade their children and families to go visit or develop cordial terms with them. Their immediate neighbors were mostly migrant men or laborers, who during the day were busy earning a living for themselves and would come home only to spend the night. There was no amiable relation between them what so ever. Most of the Zananas denied knowledge about their neighbors, and did not seem very interested in them, as one of the respondent stated: *“I live in my own world. The only relationship I like having is with my friends and my lovers (clients). If one of my neighbors wants to have me, he would have to pay my price”.*

People living alongside these dwellings see them as a nuisance to the pious Islamic way of living. Some of them even go to the point of ridiculing them so that they would leave the area. Some of the respondent claim that they have been beaten up or verbally abused when they step out of their homes. One of the Zanana recalled: *“I remember when I shifted to my current home, there were many Zananas who were doing business here, but now most of them have left, because the ‘molvi sahab’ (religious leader) instructed his followers to beat up any Zanana they saw. The followers weren’t brave enough to do so in public but did resort to throwing stones at our doors and giving us threats. They even set loose dogs on one of my fellow Zanana. She was seriously injured”.*

### Contact with the original families: loved ones left behind

Majority of the respondents interviewed had no or minimal contact with their original families. This trend was more prevalent in the Zananapan school of thought. When questioned about their original families, most of the Zananas denied having visited their family members in the past year. They did, however, admit to the fact that they missed their family members and sometimes tried contacting them on the phone. One of the respondents, rather sadly recalled: *“I loved playing with my sisters when I was a child, I had three of them and they loved me too. But now it has almost been 15 years since I last saw them. I have tried to contact them numerous times, but they never answer my calls. I just wish to see them only once before I die”.*

Khusrapan school of thought, on the other hand, claimed to be in full contact with their original families and relatives. Some of them even had their siblings visit them frequently at their homes. One of the gurus of the Khusrapan school of thought said*: “I am not ashamed of who I am. My family members realize the fact that I am different but they still love me. My younger brother got married last year and he invited me to his wedding. He also came to visit me with his wife once. I am thankful to God to have given me such a supportive family”.*

### Exclusion from occupational opportunities: selling sex, loosing respect

The role of Hijras in our community has evolved over the years. Starting from the Mughal empire where they were keepers of the ‘Harem’s’ to the current day scenario where they sell their bodies for a source of living. This transition, to some extent, is not yet complete. One can still find the remnants of the old culture scattered across the population. These hijra’s identify themselves as followers of ‘Khusrapan’. As stated earlier, this group does not indulge in commercial sex work. Their major source of income is alms and charity. Some of the Khusras even find this degrading for them and would rather prefer earning a living by their own hand. One of our respondents ran a hair cutting salon for ladies in the city and was proud of it. Some of them did tailoring on a small scale while others had tried their hand in salesmanship. But there were many accounts of Hijras being disapproved of doing proper jobs for the excuse of them being inappropriate, as stated by a respondent: *“I never wanted to be a sex seller. In the start, when I left home I tried to work in a small barbershop in my village. But I was forced to leave because the owner of that shop tried to molest me. Then I came to the city and started working as a helper in a ladies salon. The owner was a very friendly old lady who later on took me in as her apprentice. I am very grateful to her as it’s because of her that I can earn a fair living for myself”.*

Zananas showed a completely different perspective. According to them, our society itself would never let any Khusra earn a fair living, according to a Zanana*: “Men will always see Khusras as sex symbols and would want to have sexual relations with them. They see us as a cheap source of entertainment. Even when we go out to buy food, they think that we are out to gather clients. How can we work in such a hostile environment? My guru explained this to me when I was very young and I have learnt with experience that he was right”.*

Commercial sex work is not the only occupation that Zananas have. They are also famous for their dances. Many Zananas dance as a hobby and are especially invited to weddings and other ceremonies to dance for the amusement of the guests. These arrangements are very productive financially for the Zananas as they are paid heavily for them. One of the Zanana respondents tried to justify this claim by saying: *“I don’t enjoy having sex with strangers. I prefer dancing at weddings as dancing is the love of my life. But sometimes my financial conditions get so bad that I have no other choice but to sell sex”.*

### Future aspirations: chained dreams

Most Hijras from the Khusrapan way of life believe in a bright future and have made up targets to achieve in the form of religious and social achievements. One respondent said that *“I have always dreamed of performing Hajj (Holy Pilgrimage to Mecca) and I am also planning to adopt a child. I just love children, they make me smile”.* On the contrary, most Zananas considered their future to be very unpredictable and bleak. They were full of tragic events about people similar to them. Many thought that being deprived of an actual family was the root cause of these problems. In our conversation with an elderly abandoned guru whose Chelas had left him, he responded as: *“Back in the days when I used to be young, everyone knew me, I had many lovers but as I grew old all my Chelas left me one by one, I don’t know where my real family is, I sleep on an empty stomach on many a nights. It is like the only thing I expect of my future is death”.*

### Death in isolation: going away quietly

In the Pakistani society Death is a ceremony, starting from the final moments of the life of the deceased and going on for forty days. Even after that, the death anniversaries of loved ones are celebrated year after year, without missing visits to the graveyards on special occasions. Contrary to these socially acknowledged events of death and beyond, the Hijra community performs the death ceremonies of their fellows in a very secretive manner. The funerals are carried to the graveyards in the middle of the night when everyone else in the vicinity is fast asleep. These ceremonies are carried out quietly in the dark of the night with no or minimal attendance from neighbors or other acquaintances. Some Hijras mostly belonging to the Zananapan way of thought even go to the extent of defining death of a fellow as an occasion to celebrate, because the life on this earth was tough and judgmental, while the life beyond would be fair and without hurtful experiences, for God does not hurt his creatures like people do.

## Discussion

While conducting this qualitative study we have tried to get the most intimate information with regards to the social role of the Hijra community in Pakistan. It is evident from our findings that most hijras are socially excluded from performing a role of normalcy according to the prevalent social norms. They face social exclusion at all phases of their lives starting from early childhood to old age. Keeping the results in mind it is not hard to understand that because of the social exclusion faced by the hijras in terms of educational, occupational and social opportunities they are left with no other means of earning a living but to sell sex. The two schools of thought namely “Khusrapan” and “Zananapan” identified as a result of our study also delineates a clear contrast between the one group who are held in high esteem and refrain from commercial sex while the zananas (mostly men) are left with no option besides commercial sex work and beggary.

Social exclusion is a self-explanatory term, which refers to people or certain cohorts being shunned off by the society, from performing the normally expected social roles. According to Hilary Silver and S.M. Miller poverty does not only pertain to the absence of physical or monetary assets but rather includes deprivation from basic amenities essential for performing a socially viable and productive role in the community [22]. Gordon David and Peter Townsend put forth the following indicators of social exclusion; financial difficulties in the household, unaffordability of some basic needs, unaffordability of consumer durables, disadvantageous housing conditions, poor health: life expectancy; self-perceived health status, Infrequent contacts with friends and relatives, dissatisfaction with work or main activity [23]. Keeping these indicators in mind we have categorically explained how the Hijra community in Pakistan is socially excluded and how this trend has pushed them into following risky behaviors with regard to commercial sex work.

### Financial difficulties in the household

Most Hijras that we came across belonged to a poor socio-economic background, with many siblings. The issue of financial conundrums usually shifts the attention from the individual problems of the children towards more basic needs of financial viability. Most of the parents of our respondents, having gained the knowledge about the sexual deviance of their child refrained from seeking professional help, based on the fear of stigmatization, which consequently led to the barring of these children from normal societal practices. Had these parents shown more moral support towards their children, they might have ended in a better situation than the current one. The practice of regarding any sorts of gender deviances with a negative attitude in the pursuit of being masculine [9] is further supplemented by the values of the male-dominated society of Pakistan [24]. It is a common practice in Pakistan that when there are too many children to feed some of them are sent to Madrasas (religious boarding schools) [25], similarly children exhibiting sexual confusions eventually end up with the hijra community because the feeling of their absence is many a times comforted by the fact that now *“we have one less mouth to feed”.*

### Unaffordability of some basic needs

Hijras live in closed communes which act as individual family units; with the Guru serving the role of decision maker and the Chelas working as the income generators. Due to the rapid increase in inflation during the Past few decades, especially in low and middle income countries like Pakistan, the cost of running a complete household has increased substantially [26]. In this constant struggle for economic sustainability the Hijras need to resort to the idea of Easy money through commercial sex work [27]. The persistently rising rents and bills and the exponential rise in the prices of basic commodities force the Hijras to sell sex as a means of living. This point of discussion becomes even more pertinent when we look into the fact that most of the times these people are socially excluded from availing educational and occupational opportunities.

### Unaffordability of consumer durables

Originally, occupational trends of hijras leaned more towards dancing and performing art, which when displayed at public gatherings, generated enough income for them to sustain themselves [3]. With the recent hike in the prices of commodities of daily living, the general trend toward calling the Hijras to public gathering has decreased, as more and more people don’t have enough money to afford their services. This has made acquisition of consumer products, such as clothes and accessories, out of reach for the hijra community. Due to this, the hijra community started looking for alternative means of income, such as commercial sex work.

### Disadvantageous housing conditions

Commercial sex work is regarded as a taboo in most countries, with most of it taking place in secrecy [1]. Those who can afford, especially female sex workers, live in relatively better accommodations. Hijras are usually regarded as providers of ‘*Cheap Sex*’ [28], and are mostly seen living in ragtag single rooms alongside commercial areas. These rooms often don’t even have an attached bathroom or a kitchen. Some of these accommodations are at times seen to accommodate 4–5 hijra’s. Followers of Khusrapan, although being comparatively better of financially, exhibit similar housing environment, with overcrowding being the most prominent feature. This, factor along with the fact that they are socially excluded [13], adds up to the element that the hijra community is at a greater risk for acquiring and spreading sexually transmitted diseases through commercial sex trade.

### Poor health-life expectancy; self-perceived health status

Many hijra’s strongly believe that they are incomplete beings. A woman’s soul trapped in a man’s body to be precise. This in addition to the circumstance that the society shuns them away from almost every aspect of a normal living, forces them to adopt behaviors that are generally considered unhealthy. Most of our respondents failed to visit a health care professional, mainly because they thought that doctors would never take them seriously and would mock them as everyone else does. For this reason most respondents choose private practitioners of alternative medicine for treatment of any ailments that they might suffer from.

### Infrequent contacts with friends and relatives

Relatives and family member play a very crucial role in the personality development of an individual. Lack of family support can lead to many psychological and social problems. It has been vastly observed by many researchers that many Hijras lack contact with their original families [29]. Most of our respondents belonging to the Zananapan school of thought reported minimal contact with their original families. They thought that their families were ashamed of their existence and would never accept them as who they are. When probed deeply, the hijra’s accepted that the reason for this attitude is mostly commercial sex work. Followers of the Khusrapan school of thought, on the other hand, enjoyed full support of their families. The main reason for this behavior, according to them was the fact that they never do anything dubious. Lack of support from family members can be one of the reasons why the hijra’s, especially belonging to the Zananapan school of thought, propelled into the vicious cycle of social exclusion leading them to high risk behaviors like commercial sex work and drug abuse.

### Dissatisfaction with work or main activity

Gender roles in the Pakistani community like many other developing conservative societies, are well defined [30]. With most of the population still following centuries old tradition, hijra’s find themselves in a very unique situation. They cannot go out and work like other men of the community; mainly due to the fear of being ridiculed and sexually harassed. They also cannot sit home and do household chores like women, because their families see them as men and expect then to support them financially. As a majority of hijra’s today live alone or with their own kind, they find themselves antagonized by the community at general. The main reason being, that our community fails to define a role for them; a clear proof of this fact is that since the independence of Pakistan in 1947, they had not been allowed to have National Identity Registration according to their gender identification. It has only been a few years since the Supreme Court ordered the relevant authorities to register Hijras as Pakistani citizens with a right to vote [31]. Owing to poverty and the struggle of sustenance, this cohort has no other option but to turn to commercial sex work for a living.

#### Methodological considerations

The trustworthiness and credibility of the data was ensured by multiple interview sessions with the respondents. The participants of this study were identified and contacted through our gate keeper, who was also a member of the Hijra community; this substantially increased the level of trust between the participants and the research team. It is vital to understand that when it comes to conducting research on the way how various marginalized groups of people live, the information gleaned can be contextual at times. The current study could have involved a larger number of study participants in order to reach an even broader perspective, but due to financial restraints this could not be done. The authors intend to build on the discussion that will be generated by the publication of this research.

## Conclusion

Social exclusion is one of the many factors that are pushing the hijra community towards commercial sex work. We must learn to tolerate and understand the fact that every person is different, and has a right to avail the opportunities essential for a socially productive life. It is imperative to realize that almost always the social exclusion of certain groups of people has its implications of the society as a whole, in the long run. Hijra sex workers have a major role in spreading HIV and other sexually transmitted diseases. It is very important to accommodate this group into the mainstream society so that the element of social exclusion can be eliminated. Only then can any intervention, aimed at the elimination of these high risk behaviors, be effective.

## Competing interests

The authors declare that they have no competing interests.

## Authors’ contributions

MA and ZB were the main authors of the manuscript and involved in all aspects of the study. NY, ZF and AS were involved in the development of study questions based on the original idea, data collection and data analysis. BK and AD were involved in the data collection, liason with the gate keeper and data analysis. All co-authors have seen and approved the final version of the paper and have agreed to its submission for publication. All authors read and approved the final manuscript.

## Authors’ information

MA is a senior Instructor at the Department of Community Health Sciences of Shifa College of Medicine. NY is an Instructor in the Department of Basic Health Sciences of Shifa College of Medicine. ZB, BK, ZF, AS and AD are students of Final year MBBS at Shifa College of Medicine, Islamabad.

## Pre-publication history

The pre-publication history for this paper can be accessed here:

http://www.biomedcentral.com/1472-698X/12/32/prepub
